# Automated sleep stage and event detection algorithms using quality-controlled polysomnography annotations

**DOI:** 10.1093/sleepadvances/zpag054

**Published:** 2026-05-12

**Authors:** Michiru Kaneda, Sho Ogaki, Tomoyuki Nohara, Syuhei Fujita, Naoshi Osako, Tomoko Yagi, Yasuhiro Tomita, Takanori Ogata

**Affiliations:** ACCELStars, Inc., Tokyo, Japan; ACCELStars, Inc., Tokyo, Japan; ACCELStars, Inc., Tokyo, Japan; ACCELStars, Inc., Tokyo, Japan; ACCELStars, Inc., Tokyo, Japan; School of Medical Technology, Kurume University, Fukuoka, Japan; Sleep and Respiratory Medicine, Toranomon Hospital, Tokyo, Japan; Graduate School of Medicine, The University of Tokyo, Tokyo, Japan; Faculty of Medicine, Juntendo University, Tokyo, Japan; ACCELStars, Inc., Tokyo, Japan

**Keywords:** polysomnography, sleep stage classification, arousal detection, respiratory event detection, inter-scorer agreement, automated sleep analysis

## Abstract

**Study Objectives:**

To develop machine learning models for sleep stage classification, arousal detection, and respiratory event detection from overnight polysomnography, and to evaluate their performance relative to expert scorers.

**Methods:**

Overnight polysomnography recordings were obtained from healthy participants and participants referred for suspected sleep-disordered breathing. Four certified scorers completed calibration sessions and generated reference annotations for sleep stages, arousals, and respiratory events. A subset of recordings was independently annotated by all scorers to support consensus analyses, enabling direct comparison between model outputs and human inter-scorer agreement. Gradient-boosted decision tree models were trained using hand-crafted features derived from standard physiological signals.

**Results:**

Sleep stage classification achieved an accuracy of 0.840, a Cohen’s kappa of 0.791, and an F1-score of 0.841, with limits of agreement for total sleep time of approximately ±0.5 h. Arousal detection achieved an F1-score of 0.733, with limits of agreement for the arousal index of approximately ±15 events/h. Respiratory event detection achieved an F1-score of 0.818, with limits of agreement for the apnea–hypopnea index also within approximately ±15 events/h. In consensus analyses, model performance was comparable to human inter-scorer agreement for sleep stages and arousals, while remaining below human inter-scorer agreement for respiratory events, despite high absolute performance relative to prior studies.

**Conclusions:**

The proposed models achieved performance approaching human-level agreement across major sleep scoring tasks. These findings indicate that high consistency in expert annotations is a key factor underlying robust model performance and support the use of quality-controlled annotations for developing reliable automated sleep analysis systems.

**Statement of Significance**

Manual scoring of overnight sleep studies remains a major bottleneck in sleep medicine, limiting efficiency, consistency, and large-scale research. This study demonstrates that interpretable automated analysis can achieve performance approaching human-level agreement for core sleep scoring tasks when reference annotations are highly consistent. By directly comparing model outputs with calibrated inter-scorer agreement, the results show that annotation quality is a key determinant of attainable accuracy, rather than model complexity alone. Such systems may provide stable and reproducible reference outputs that support clinical decision-making, scorer training, and standardization across centers. Important remaining challenges include validation across institutions and populations, robustness to real-world signal artifacts, and extension to clinically meaningful subtypes of respiratory events.

## Graphical Abstract

**Graphical Abstract:**
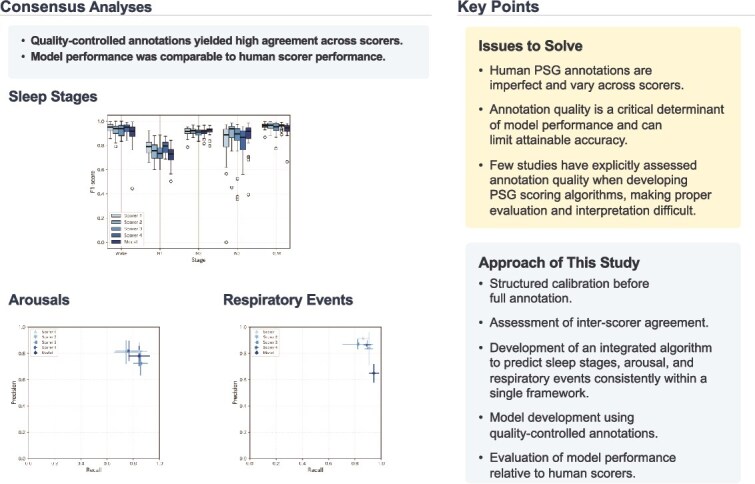

## Introduction

Sleep disorders such as insomnia and sleep-disordered breathing (SDB) are highly prevalent conditions associated with increased cardiovascular, metabolic, and neurocognitive risks [1–4]. Accurate evaluation of sleep architecture and respiration is therefore essential for diagnosis and management, with polysomnography (PSG) remaining the gold standard modality [5, 6]. By capturing comprehensive physiological signals, PSG enables precise assessment of sleep physiology and disease severity, supporting optimal clinical and therapeutic decision-making [7].

PSG typically records multiple physiological channels, including electroencephalography (EEG), electrooculography (EOG), electromyography (EMG), electrocardiography (ECG), respiratory effort, airflow, and oxygen saturation. From these data, trained scorers manually annotate events such as sleep stages, arousals, and respiratory abnormalities [8]. The American Academy of Sleep Medicine (AASM) manual provides standardized scoring rules that are widely adopted across sleep laboratories [9–11].

Although these criteria have improved standardization, PSG scoring remains predominantly based on visual inspection by trained scorers. This process is labor intensive, time consuming, and dependent on specialized expertise, often limiting diagnostic throughput. Because PSG interpretation inherently depends on human judgment, scoring accuracy is constrained by perceptual and cognitive limitations, resulting in notable inter-scorer variability even under AASM guidelines [12–16]. Variations in institutional practices, scorer training, and interpretation further challenge standardization, limiting both clinical consistency and research reproducibility. Consequently, there has been increasing interest in automated PSG analysis to streamline clinical workflows, reduce scoring burden, and improve inter-scorer reliability.

Earlier studies employed feature-based machine learning approaches such as support vector machines, random forests, and decision trees for automatic sleep stage classification [17–22]. Subsequent works introduced deep learning architectures capable of learning temporal and spectral representations directly from raw data, achieving improved accuracy [23–27]. More recently, interpretable modeling approaches have emerged, including image-based representations and cross-modal transformer architectures designed to provide human-aligned explanations of PSG predictions [28–30]. At the same time, traditional machine learning pipelines have been shown to perform comparably to state-of-the-art deep learning models [31].

Similar progress has occurred in automated detection of arousals and respiratory events. Early automated approaches relied on hand-crafted features derived from EEG, ECG, airflow, and respiratory effort [32–35], whereas later research increasingly adopted deep learning models that directly capture temporal dependencies and event durations from PSG signals [36–40].

Furthermore, a recent hybrid pipeline integrates deep learning and signal processing to analyze sleep staging, arousal, and respiratory events, and additionally detects limb movements within the same framework [41].

Many of these studies rely on publicly available PSG databases, which provide valuable benchmarks for cross-study comparison. However, these datasets differ considerably in their scoring conventions and preprocessing standards. Older databases were annotated according to the Rechtschaffen and Kales criteria [42], and although conversion guidelines to the AASM system exist [43], they are not perfectly equivalent. Moreover, studies vary in how they select and segment data—such as the definition of lights-out and lights-on intervals, the treatment of high-noise segments, and the allocation of data into training and test sets—as well as in how they match events for evaluation. Because these methodological details are not standardized across datasets or studies, direct numerical comparison of reported performance metrics is inherently difficult.

In addition to these inconsistencies, the reliability of human-generated annotations themselves remains uncertain. Several studies have investigated uncertainty in sleep stage scoring and compared the performance of automated systems with that of human scorers [41, 44, 45]. These studies have demonstrated that substantial uncertainty exists in manual scoring, and that automated models can achieve levels of agreement comparable to trained experts and, in some cases, exceed the consistency of individual scorers relative to a selected reference standard.

Importantly, recent work has emphasized that disagreement among human scorers often reflects intrinsic ambiguity in sleep stage transitions rather than annotation errors alone. In this context, probabilistic sleep staging frameworks have been proposed to characterize such uncertainty by assigning a distribution over sleep stages to each epoch, thereby providing a quantitative description of scorer disagreement and transitional physiology beyond conventional hard labels [46–48]. Collectively, these findings indicate that both human agreement and apparent model performance are constrained by the inherent uncertainty of PSG scoring, and that improvements in automated analysis should be interpreted in relation to annotation reliability and ambiguity.

In the present study, a high-quality reference dataset was established through a structured calibration process rather than through adjudicated consensus labeling. Multiple certified sleep scorers first participated in targeted calibration sessions to harmonize their scoring criteria for sleep stages, arousals, and respiratory events. After calibration, each scorer independently annotated PSG recordings under unified scoring guidelines, yielding a set of consistent and quality-controlled labels suitable for algorithm development. A subset of recordings was double scored to quantify inter-scorer reliability, which was then compared with model performance to assess the extent to which the automated system approximated human-level agreement. This design avoids the variability introduced by uncalibrated datasets while providing a set of consistently generated, high-quality annotations for model development and evaluation.

Using this rigorously standardized annotation set, a gradient-boosted decision tree (GBDT) [49]-based framework was developed and evaluated for sleep stage classification, arousal detection, and respiratory event detection. Model performance was assessed relative to multiple expert scorers to provide a benchmark grounded in high-quality, consistently generated PSG annotations.

## Materials and methods

### Ethics statements

This study was approved by the Ethics Committee of Medical Corporation Kouseikai Sone Clinic (Protocol IDs: SUG08869 and SUG08884) and by the Ethics Committee of Koga Hospital 21 (Protocol ID: 21-033). Written informed consent was obtained from all participants in the prospective studies at Sone Clinic. For the retrospective study at Koga Hospital 21, the requirement for written informed consent was addressed through an opt-out process implemented by the hospital and disclosed on its website, in accordance with the approved protocol. All data were anonymized prior to analysis, and no identifying information is included in this manuscript.

### Dataset

Three types of overnight PSG datasets were used in this study. The first dataset consisted of PSG recordings acquired from healthy participants in a dedicated in-house sleep research laboratory equipped for controlled overnight monitoring, with SOMNO BT Plus (SOMNOmedics AG) devices. The second and third datasets consisted of PSG data obtained at two different clinical sleep centers referred for evaluation of suspected SDB, primarily obstructive sleep apnea. Alice 6 LDx (Royal Philips) and SOMNOtouch RESP (SOMNOmedics AG) devices were used to acquire PSG data at the two clinical centers, respectively. All PSG recordings were obtained using standard clinical PSG montages in accordance with the AASM Scoring Manual.

For the first and second datasets, six EEG channels recommended by the AASM manual for sleep staging were included: F3–M2, F4–M1, C3–M2, C4–M1, O1–M2, and O2–M1. The third dataset included only three EEG channels, F3–M2, C3–M2, and O1–A2. All datasets included two EOG channels, E1–M2 and E2–M1 and one chin EMG channel, as well as one nasal pressure airflow channel and one SpO_2_ channel. Additional clinical PSG channels were also recorded as part of a standard clinical PSG montage, including thermistor airflow, thoracoabdominal effort signals, and bilateral anterior tibialis EMG. These channels were available during manual scoring, but only the channels used for feature extraction are described here in detail.

Although the third dataset has only three EEG channels, it is enough to perform manual analysis if all three channels are clear. On the other hand, this study focuses on the development with full six EEG channels, and the third dataset was used only for scorer calibration, not for model development or evaluation.

In the following tables, the term “Healthy” refers to the first dataset of PSG recordings from healthy participants, while “SDB” refers to the second dataset of PSG recordings from participants with suspected SDB. The third dataset, which was used only for scorer calibration, is referred to as “Calibration SDB.”

Table 1 summarizes the sampling frequencies of the PSG channels from which features were extracted.

**Table 1:** Sampling Frequencies (Hz) of PSG Channels

Channel	SOMNO BT Plus	Alice 6 LDx	SOMNOtouch RESP
Electroencephalography	256	500	256
Electrooculography	256	500	256
Electromyography	256	500	256
Nasal pressure airflow	256	100	256
SpO_2_	4	1	4

For the model development and evaluation, a total of 604 recordings from healthy participants and 245 recordings from participants with suspected SDB were initially obtained as the first and second datasets, respectively. Recordings were screened to include only complete overnight sessions with sufficient signal quality and no major signal loss. For participants with multiple recorded nights, a single night was selected to avoid duplication at the participant level. After this quality control, 473 healthy recordings and 244 SDB recordings were included in the sleep stage classification study (Table 2), as these recordings were sufficiently clear to allow full night sleep stage classification. From this quality-controlled subset, 412 healthy recordings and 178 SDB recordings were further included in the arousal detection study, reflecting the additional requirement that data quality be sufficient over the entire night for reliable arousal and respiratory event detection (Table 3).

**Table 2:** PSG Datasets for the Sleep Stage Study

Characteristic	Healthy	SDB
Recording period	May 4, 2022–September 30, 2024	August 1, 2023–June 24, 2024
*N* participants	473	244
Sex, F/M (%)	44.2/55.6^*^	21.3/78.7
Age (years)	33.4 (13.3)	56.7 (12.2)
	[9, 59]	[24, 85]
BMI (kg/m${\kern0em }^2$)	21.6 (3.1)	25.5 (4.3)
	[15.6, 37.4]	[16.1, 45.6]
TST (h)	6.2 (1.4)	6.0 (1.0)
	[1.5, 9.8]	[2.3, 7.8]

^*^One participant did not answer.

BMI: body mass index; TST: total sleep time.

The Healthy group refers to participants without suspected sleep disorders. The SDB group includes participants referred for evaluation of suspected SDB.

For age, BMI, and TST, numbers are presented as mean (*SD*) [range].

**Table 3:** PSG Datasets for the Arousal and Respiratory Event Studies

Characteristic	Healthy	SDB
Recording period	May 4, 2022–September 30, 2024	August 1, 2023–June 24, 2024
*N* participants	412	178
Sex, F/M (%)	44.4/55.6	19.7/80.3
Age (years)	33.5 (13.2)	56.5 (12.4)
	[9, 59]	[24, 85]
BMI (kg/m${\kern0em }^2$)	21.6 (3.1)	25.2 (4.3)
	[15.6, 32.3]	[16.1, 45.6]
TST (h)	6.2 (1.4)	6.0 (1.0)
	[1.5, 9.8]	[2.3, 7.8]
ArI (1/h)	14.8 (9.1)	41.1 (17.6)
	[2.1, 72.5]	[6.2, 114.5]
AHI (1/h)	–	40.9 (20.6)
		[3.5, 103.0]

BMI: body mass index; TST: total sleep time; ArI: arousal index; AHI: apnea–hypopnea index.

The Healthy group refers to participants without suspected sleep disorders. The SDB group includes participants referred for evaluation of suspected SDB.

For age, BMI, TST, ArI, and AHI, numbers are presented as mean (*SD*) [range].

For the respiratory event detection study, only recordings from participants with suspected SDB were used.

For the evaluation of inter-scorer agreement, 27 healthy recordings were used in the consensus study for sleep stage classification and arousal detection, and five SDB recordings were used in the respiratory event consensus study (Table 4).

**Table 4:** PSG Datasets for the Consensus Studies

Characteristic	Healthy	SDB
Recording period	May 4, 2022–October 29, 2022	August 1, 2023–August 16, 2023
*N* participants	27	5
Sex, F/M (%)	29.6/70.4	0.0/100.0
Age (years)	40.1 (11.5)	56.8 (13.6)
	[23, 59]	[39, 73]
BMI (kg/m${\kern0em }^2$)	22.1 (2.0)	24.9 (4.7)
	[18.8, 26.0]	[20.3, 32.5]
TST (h)	6.7 (1.2)	5.5 (0.5)
	[4.8, 9.5]	[5.1, 6.1]
ArI (1/h)	13.3 (5.3)	19.7 (7.6)
	[2.6, 26.2]	[12.9, 29.4]
AHI (1/h)	–	32.1 (16.7)
		[13.4, 51.2]

BMI: body mass index; TST: total sleep time; ArI: arousal index; AHI: apnea–hypopnea index.

The Healthy group refers to participants without suspected sleep disorders. The SDB group includes participants referred for evaluation of suspected SDB.

For age, BMI, TST, ArI, and AHI, numbers are presented as mean (*SD*) [range].

Table 5 summarizes the PSG recordings used for scorer calibration. For sleep stage and arousal scoring, calibration was conducted using 30 recordings from healthy participants and 7 recordings from the Calibration SDB group. Of the 30 healthy recordings, 25 were included in the consensus subset used in this study. The remaining five were not included in model development or evaluation: three were excluded because they were duplicate recordings from participants already represented elsewhere in the dataset, and two were excluded because part of the recording contained noise that made scoring impossible in some intervals. In addition, two further healthy recordings were later scored by all four scorers for monitoring purposes and were included in the consensus subset.

**Table 5:** PSG Datasets Used for Scorer Calibration

Characteristic	Healthy	SDB	Calibration SDB
Recording period	May 4, 2022–September 9, 2022	August 1, 2023–August 16, 2023	November 21, 2019–May 6, 2022
*N* participants	30	5	7
Sex, F/M (%)	26.7/73.3	0.0/100.0	14.3/85.7
Age (years)	39.8 (10.4)	56.8 (13.6)	63.3 (10.8)
	[23, 59]	[39, 73]	[46, 74]
BMI (kg/m${\kern0em }^2$)	22.2 (1.9)	24.9 (4.7)	27.9 (4.2)
	[19.6, 26.0]	[20.3, 32.5]	[23.6, 33.9]
TST (h)	6.7 (1.1)	5.5 (0.5)	7.2 (1.1)
	[4.8, 9.5]	[5.1, 6.1]	[5.2, 8.4]
ArI (1/h)	12.8 (4.9)	19.7 (7.6)	18.8 (3.9)
	[2.6, 22.6]	[12.9, 29.4]	[12.4, 24.0]
AHI (1/h)	–	32.1 (16.7)	25.8 (11.7)
		[13.4, 51.2]	[12.9, 40.3]

BMI: body mass index; TST: total sleep time; ArI: arousal index; AHI: apnea–hypopnea index.

The Healthy group refers to participants without suspected sleep disorders. The SDB group and the Calibration SDB group include participants referred for evaluation of suspected SDB. The calibration SDB group was used only for scorer calibration.

For age, BMI, TST, ArI, and AHI, numbers are presented as mean (*SD*) [range].

For respiratory event calibration, five recordings from the SDB group and seven recordings from the Calibration SDB group were used. The five SDB recordings were the same subset as those used in the respiratory event consensus study.

### Annotation

Annotation of sleep stages, arousals, and respiratory events followed the AASM Scoring Manual. While the current manual is Version 3 [9], scoring in this study was conducted according to Version 2.6 [50], which was the version routinely used in Japan at the time. For each recording, the lights-out and lights-on times were first identified, and scoring was performed only within the interval between lights-out and lights-on.

Annotation was performed by four experienced scorers, each affiliated with a different institution. For sleep stage scoring, scorer calibration was conducted using recordings from the Healthy group together with recordings from the Calibration SDB group, as summarized in Table 5. The target was to achieve at least 85 per cent agreement with the lead scorer for each sleep stage in each recording, while for recordings from the Calibration SDB group, the minimum target agreement was set at 80 per cent. Each scorer first independently scored the calibration recordings. Segments with substantial disagreement were then reviewed with the lead scorer, and recordings with frequent disagreements were selectively rescored, with up to approximately four rounds of rescoring performed when needed.

For arousal scoring, scorer calibration was conducted using the same calibration recordings, and the resulting agreement was considered sufficient for the purposes of this study.

For respiratory event scoring, calibration was performed with a target event-level agreement of approximately 80 per cent with the lead scorer. After an initial round of scoring, discrepant events were reviewed with the lead scorer, and depending on the recording, rescoring was repeated approximately three or four times.

After calibration, the remaining PSG recordings were distributed among the scorers, and each scorer independently annotated the assigned recordings. Two additional healthy recordings were also scored by all four scorers for sleep stage and arousal scoring and were included in the consensus dataset.

Except for the consensus subset, all respiratory event annotations were performed by a single expert scorer. Respiratory events included hypopnea, obstructive apnea, central apnea, and mixed apnea; however, for the purposes of this study, all were treated collectively as a single class of respiratory events.

Because PSG data were collected over an extended period, annotation sessions were also conducted at different times, which may have introduced minor temporal variations in scoring style.

### Algorithms

This study employed a GBDT-based framework for three tasks: sleep stage classification, arousal detection, and respiratory event detection. All tasks were formulated as epoch-wise classification problems based on manually annotated PSG signals. Data were segmented into fixed length epochs: 30-s epochs for sleep stage classification and 1-s epochs for arousal and respiratory events. From each epoch, task-specific sets of time domain and frequency domain features were extracted from EEG, EOG, EMG, nasal pressure airflow, and SpO_2_ signals. Although the PSG recordings were acquired at different native sampling frequencies (Table 1), the feature extraction procedures operated on time-normalized segments and predefined frequency bands, ensuring that signals from both devices yielded harmonized feature representations.

A hierarchical model structure was used: the estimated sleep stage from the sleep stage model was included as a feature for arousal detection, and both the estimated sleep stage and arousal labels were used as features for respiratory event detection. To capture temporal dependencies in physiological signals, features from the target epoch and neighboring epochs were concatenated to form an extended context window.

All models were implemented using LightGBM [51], chosen for its suitability for heterogeneous tabular features and efficient training. For arousal and respiratory events, the 1-s epoch-wise probabilities output by the classifier were merged using task-specific post-processing procedures to reconstruct contiguous events.

The following subsections describe the task-specific feature sets, temporal context structures, classification models, and post-processing steps. Throughout all tasks, airflow features refer specifically to nasal pressure airflow derived from a cannula sensor, which was the airflow modality used in this study.

### Sleep stage classification

#### Sleep stage feature extraction

Data were segmented into consecutive, non-overlapping 30-s epochs. For each epoch, a total of 1450 features were computed from EEG, EOG, and EMG channels.

For the EEG signals (six channels), power spectral density (PSD) features were computed using the Welch method [52], which estimates the power spectrum by averaging modified periodograms of overlapping signal segments to reduce variance. Each 30-s epoch was divided into six 5-s subsegments, and the log PSD was estimated for the following frequency bands: slow delta (0.5–2 Hz), fast delta (2–4 Hz), theta (4–8 Hz), alpha (8–13 Hz), beta (13–30 Hz), and spindle (11–16 Hz). Band power ratios were also derived. Identical PSD and ratio features were computed from the PSD estimated over the full 30-s epoch. Time domain features included the mean, root mean square (RMS), skewness, kurtosis, and 0.75 quantile for each channel.

For the EOG signals (two channels), PSD features were extracted at 1 Hz resolution from 0 to 35 Hz using the same six segment structure as for EEG. Time domain statistics (mean, RMS, skewness, kurtosis, 0.75 quantile) were also computed.

For the EMG signal (one channel), RMS values were computed for each 1-s segment within the epoch, and the sequence of RMS values served as EMG features.

#### Sleep stage classification model and temporal context

To incorporate temporal information, features from the target epoch and the two preceding and two following epochs were concatenated, forming a five-epoch context window (7250 features per input sample). LightGBM was used to classify each epoch into Wake, N1, N2, N3, or rapid eye movement (REM) sleep.

### Arousal detection

#### Arousal feature extraction

For arousal detection, data were segmented into 1-s epochs, and labels were generated by converting event-based arousal annotations into 1-s epoch-wise labels.

For each epoch, 34 features were computed from EEG, EOG, EMG, nasal pressure airflow, and SpO_2_ signals, in addition to the estimated sleep stage.

The sleep stage estimated by the sleep stage model was included as a categorical variable and converted into dummy variables for N1, N2, N3, and REM.

For the EEG signals, PSD features were computed from each 1-s segment using the same spectral bands defined for sleep stage classification. From these, only beta power, spindle power, and the theta/alpha ratio were retained, based on feature importance analysis indicating their relevance for arousal discrimination. RMS was also computed for each EEG channel.

For the EOG and EMG channels, the RMS of each 1-s segment was extracted.

For the nasal pressure airflow signal, respiratory volume per time (RVT) was computed using a Hilbert-based method that estimates instantaneous respiratory amplitude and period [53], and the RVT values were then averaged within each 1-s segment.

For the SpO_2_ signal, the RMS and mean of each 1-s segment were used.

#### Arousal classification model and temporal context

Features from the target epoch and the 30 preceding and 30 following epochs were concatenated to form a 61 epoch context window (2074 features per sample). LightGBM was used to classify each epoch as arousal or non-arousal.

#### Arousal post-processing

Epochs with predicted arousal probability above 0.3 were marked as arousal candidates. Gaps of 5 s or less between candidates were bridged to form continuous events. Events shorter than 3 s were removed. Thresholds were selected by minimizing the mean absolute error between predicted and annotated arousal index (ArI).

### Respiratory event detection

#### Respiratory event feature extraction

For respiratory event detection, data were segmented into 1-s epochs, and respiratory annotations were converted into epoch-wise labels following the same procedure as for arousals. For each epoch, seven features were computed from the estimated sleep stage, estimated arousal labels, nasal pressure airflow, and SpO_2_ signals.

The estimated sleep stage was included as dummy variables (N1, N2, N3, REM). The arousal detection model provided a binary arousal indicator (1 for predicted arousal, 0 otherwise).

For the nasal pressure airflow signal, RVT was computed for each 1-s segment as described in the Arousal Feature Extraction section. For the SpO_2_ signal, the mean of each 1-s segment was included.

#### Respiratory event classification model and temporal context

A temporal context window of 61 s was constructed by concatenating features from the target epoch with the 30 preceding and 30 following epochs (427 features per input sample). LightGBM was used to classify each epoch as either a respiratory event or a non-event.

#### Respiratory event post-processing

Epochs with predicted probability above 0.7 were marked as respiratory event candidates. Gaps of 2 s or less between candidate epochs were bridged to form continuous events. Events shorter than 10 s were removed. Thresholds were optimized by minimizing the mean absolute error in apnea–hypopnea index (AHI) compared with manual annotations.

### Model training and evaluation

#### Cross-validation

All models were trained and evaluated using a participant-level five-fold cross-validation (CV) framework. Because the dataset comprised healthy individuals and participants with suspected SDB, the two groups were handled separately when constructing the folds. Within each group, participants were randomly divided into five approximately equal subsets. Five final folds were then created by pairing the corresponding healthy and SDB subsets, ensuring comparable proportions of healthy and SDB recordings in each fold.

In each CV iteration, three folds were used for training, one fold served as the validation set for early stopping, and the remaining fold was held out for testing. For arousal detection and respiratory event detection, the same participant-level fold assignments established for sleep stage classification were retained. Each participant thus had a consistent fold index (folds 1–5) across all tasks, while the actual recordings used for arousal or respiratory event detection corresponded to the task-specific subsets. This ensured consistent participant grouping across tasks and prevented information leakage when sleep stage or arousal predictions were used as input features in downstream models.

Unless otherwise specified, all performance metrics reported in the Results section were computed on the held-out test fold in each CV iteration and averaged across the five folds.

#### Sleep stage evaluation

To evaluate sleep stage classification performance, true and predicted sleep stages were compared for all 30-s epochs. From these epoch-wise labels, metrics (accuracy, Cohen’s kappa coefficient, macro averaged F1-score, and stage-wise F1-score for Wake, N1, N2, N3, and REM) were computed for each test fold and then averaged across the five folds.

At the recording level, total sleep time (TST) was calculated separately from the reference annotations and from the model outputs by summing the durations of all epochs labeled as N1, N2, N3, or REM for each recording. For each recording, the residual was defined as predicted TST minus true TST. Agreement between true and predicted TST was then summarized using residual plots and Bland–Altman limits of agreement (LoA) [54].

#### Event-based metrics

For arousal and respiratory event detection, event-based performance was evaluated by treating each true event (reference annotation) and each predicted event (model output) as a time interval defined by its onset and duration. For every pair of true and predicted events, the intersection over union (IoU) metric was computed as the ratio of the duration of their temporal overlap to the duration of their union [55]. A predicted event was considered a match if its IoU with a true event was at least 0.3. Matched pairs were counted as true positives (TP), true events without a matching prediction (IoU $\le$0.3) were counted as false negatives (FN), and predicted events without a corresponding true event were counted as false positives (FP). The IoU threshold of 0.3 was chosen to align with previously proposed evaluation protocols for respiratory event detection, allowing direct comparison with prior studies that benchmarked automated algorithms against human scorers [45].

From TP, FP, and FN, precision, recall, and F1-score were calculated for each test fold and then averaged across the five folds.

At the recording level, ArI and AHI were computed from both reference annotations and model outputs by dividing the total number of detected events by the TST in hours. For each recording, the residual was defined as predicted index minus true index.

#### Consensus annotations and human-model comparison

Consensus subsets were defined a priori for inter-scorer and human model comparison analyses. For sleep stage classification and arousal detection, 27 PSG recordings from healthy participants were annotated independently by all four scorers (Table 4). For respiratory event detection, five recordings from participants with suspected SDB were annotated by all four scorers.

For sleep stage classification, each scorer’s annotations were compared against consensus annotations derived from other scorers’ labels [56]. The model outputs were compared against consensus annotations created from the three highest accuracy scorers out of four [44].

For arousal and respiratory event detection, event-based annotations on the consensus subset were converted to 1-s epoch-wise binary labels (arousal vs. non-arousal) for each scorer. For each epoch, a majority vote among the three selected scorers (at least two arousal labels) was required to label the epoch as arousal, and consecutive arousal-labeled epochs were merged into continuous events, yielding consensus arousal annotations [36]. Each scorer’s annotations were compared against consensus annotations derived from the other scorers’ labels. The model outputs were compared against consensus annotations created from the three highest accuracy scorers out of four.

For the consensus analyses, all metrics were computed based on each recording in the consensus subset and then averaged across recordings.

## Results

### Performance metrics

#### Sleep stage classification

Table 6 summarizes the normalized confusion matrix across all folds.

**Table 6:** Normalized Confusion Matrix for Sleep Stage Classification

True/predicted	Wake	N1	N2	N3	REM
Wake	89.3	7.6	0.9	0.1	2.0
N1	6.3	68.6	19.6	0.1	5.4
N2	0.4	6.4	86.9	4.7	1.5
N3	0.1	0.1	14.4	85.4	0.0
REM	1.6	6.1	3.6	0.1	88.7

Values are expressed as row-wise percentages.

True labels (reference annotations) are shown in rows, and predicted labels (model outputs) are shown in columns.

Across the test folds, the model achieved the following performance, reported as mean (*SD*): accuracy of $0.840\ (0.006)$, Cohen’s kappa of $0.791\ (0.007)$, and an overall F1-score of $0.841\ (0.005)$. Stage-wise F1-scores for Wake, N1, N2, N3, and REM were $0.890\ (0.012)$, $0.711\ (0.004)$, $0.848\ (0.005)$, $0.865\ (0.009)$, and $0.892\ (0.006)$, respectively. These results indicate strong discrimination for Wake, N2, N3, and REM, whereas the lower performance observed for N1 is consistent with previous PSG-based classification studies.

**Figure 1:**
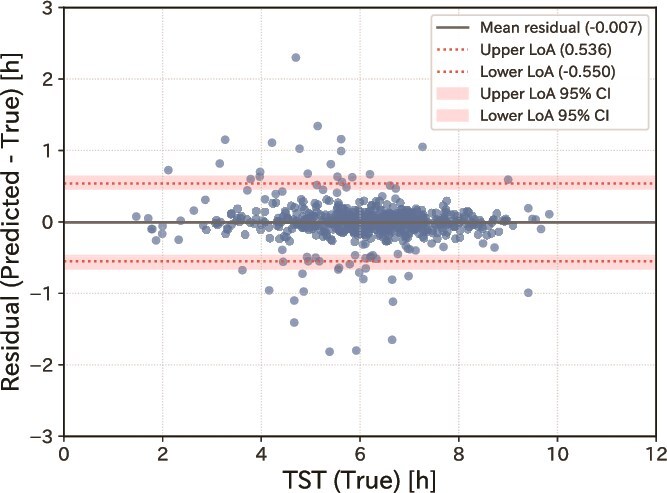
Residual plot for TST. The horizontal axis represents the true TST (from reference annotations) in hours, and the vertical axis represents the residual, defined as predicted TST (from model output) minus true TST. Each point corresponds to an individual recording. The solid horizontal line indicates the mean residual. The upper and lower dashed lines represent the LoA, calculated as the mean residual ±1.96 *SD* of the residuals. The shaded regions indicate the 95 per cent confidence intervals for the mean and LoA lines.

Figure 1 shows the residual distribution for TST estimates. TSTs were calculated for each recording by summing the durations of N1, N2, N3, and REM stages. Residuals were symmetrically distributed around zero without a systematic bias, and the LoA were approximately $\pm$0.5 h.

#### Arousal detection

Event-based performance was evaluated using the IoU-based matching criterion defined in the Model Training and Evaluation section. The model achieved mean (*SD*) recall, precision, and F1-scores of $0.725\ (0.010)$, $0.742\ (0.018)$, and $0.733\ (0.011)$, respectively. These results indicate balanced sensitivity and specificity, with stable performance across folds.

**Figure 2:**
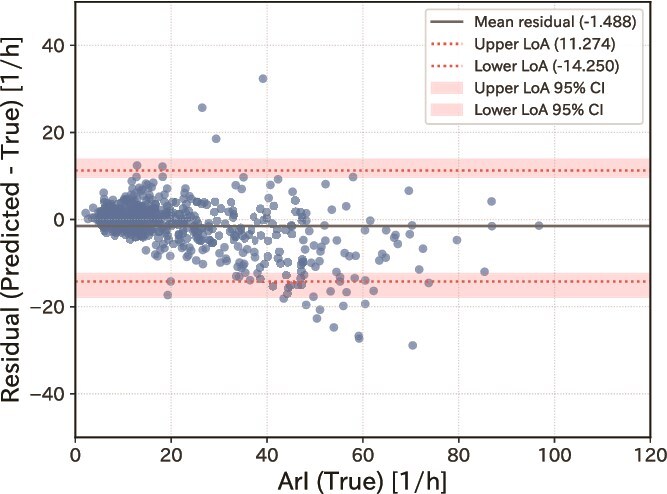
Residual plot for the ArI. The horizontal axis represents the true ArI (from reference annotations), and the vertical axis represents the residual, defined as predicted ArI (from model output) minus true ArI. Each point corresponds to an individual recording. The solid horizontal line indicates the mean residual. The upper and lower dashed lines represent the LoA, calculated as the mean residual ±1.96 *SD* of the residuals. The shaded regions indicate the 95 per cent confidence intervals for the mean and LoA lines.

Figure 2 shows the residual plot for the ArI. The LoA were approximately $\pm$15 events/h, and only a small number of outliers were observed. A slight tendency toward more negative residuals at higher ArI values may be present, although the number of observations in this range was limited.

#### Respiratory event detection

Respiratory event detection employed the same IoU-based matching and performance metrics as arousal detection. The model achieved mean (*SD*) recall, precision, and F1-scores of $0.829\ (0.010)$, $0.807\ (0.016)$, and $0.818\ (0.012)$, respectively.

**Figure 3:**
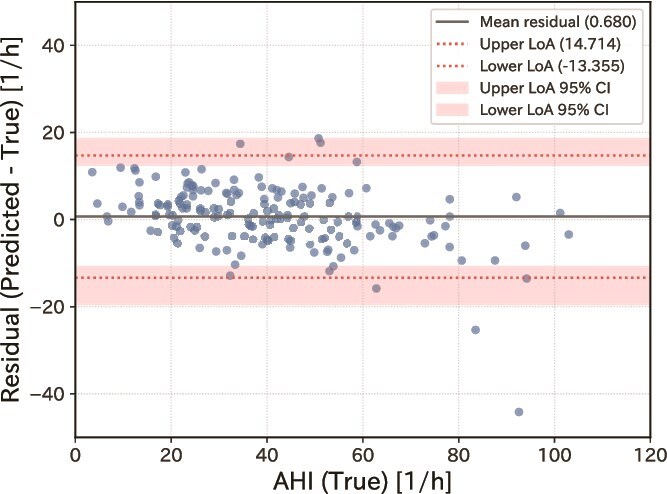
Residual plot for the AHI. The horizontal axis represents the true AHI (from reference annotations), and the vertical axis represents the residual, defined as predicted AHI (from model output) minus true AHI. Each point corresponds to an individual recording. The solid horizontal line indicates the mean residual. The upper and lower dashed lines represent the LoA, calculated as the mean residual ±1.96 *SD* of the residuals. The shaded regions indicate the 95 per cent confidence intervals for the mean and LoA lines.

Figure 3 shows the residual plot for the AHI. Residuals were centered around zero with no systematic bias across the range of AHI values. The LoA were approximately $\pm$15 events/h, and only a few outliers were observed in the high AHI range.

### Consensus study

#### Sleep stage classification consensus

**Figure 4:**
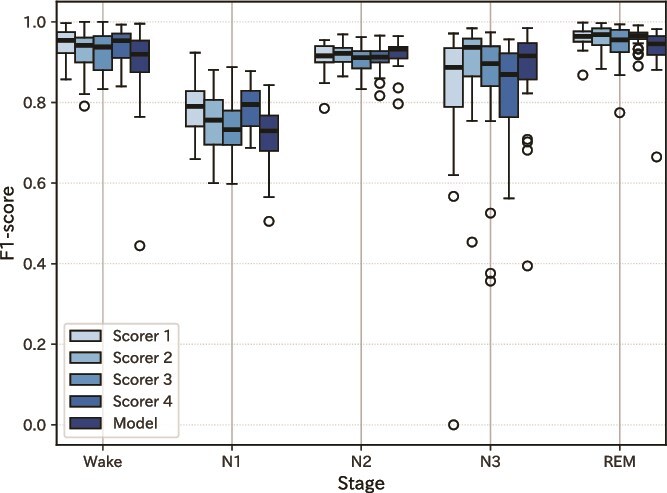
F_1_-score for each sleep stage of individual scorers and the model against consensus annotations. Boxplots showing the distribution of F_1_-score across the 27 recordings for each sleep stage (wake, N1, N2, N3, REM) are presented for each scorer and the model. For each group, the box represents the interquartile range (IQR), with the thick horizontal line indicating the median. Whiskers extend to 1.5 $\times$ IQR or to the most extreme data points within that range, and individual points beyond the whiskers are plotted as outliers.

Consensus analysis for the sleep stage classification task was performed on the 27 recording subset described in the Consensus Annotations and Human-Model Comparison section. Metrics were obtained for each recording, and means and *SD* were calculated across the recordings. Tables 7 and 8 summarize the overall and stage-wise performance metrics for individual scorers and the model against the consensus labels. Figure 4 shows the distribution of stage-wise F1-score across recordings.

**Table 7:** Performance Metrics for Sleep Stage Classification of Individual Scorers and the Model

Scorer	Accuracy	Cohen’s kappa	F_1_-score
Scorer 1	0.915 (0.030)	0.878 (0.042)	0.916 (0.029)
Scorer 2	0.916 (0.031)	0.880 (0.045)	0.917 (0.031)
Scorer 3	0.902 (0.032)	0.859 (0.046)	0.902 (0.033)
Scorer 4	0.912 (0.022)	0.874 (0.029)	0.911 (0.021)
Model	0.907 (0.040)	0.864 (0.061)	0.905 (0.042)

Values are presented as mean (*SD*) calculated from 27 recordings.

**Table 8:** Stage-Wise F_1_-Scores for Sleep Stage Classification of Individual Scorers and the Model

Scorer	Wake	N1	N2	N3	REM
Scorer 1	0.947 (0.037)	0.789 (0.061)	0.912 (0.038)	0.817 (0.199)	0.961 (0.026)
Scorer 2	0.928 (0.053)	0.751 (0.075)	0.921 (0.027)	0.890 (0.111)	0.960 (0.030)
Scorer 3	0.925 (0.052)	0.736 (0.069)	0.905 (0.036)	0.842 (0.167)	0.946 (0.048)
Scorer 4	0.942 (0.040)	0.783 (0.055)	0.911 (0.034)	0.838 (0.104)	0.961 (0.024)
Model	0.897 (0.103)	0.713 (0.078)	0.920 (0.035)	0.874 (0.127)	0.931 (0.060)

Values are presented as mean (*SD*) calculated from 27 recordings.

The model demonstrated comparable performance to that of human scorers across all sleep stages. The score for the N1 stage was lower than for other stages but still within the range of human scorer performance.

#### Arousal detection consensus

**Figure 5:**
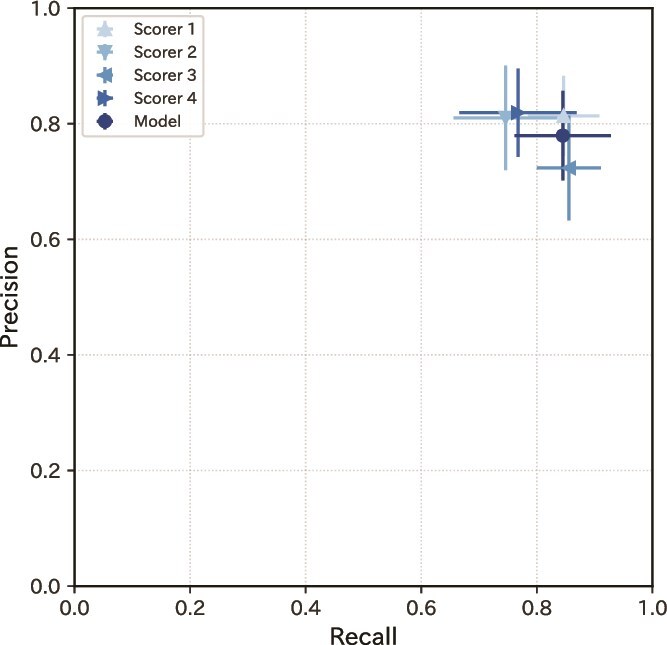
Recall and precision for arousal detection by individual scorers and by the model, evaluated against consensus annotations. Mean recall and precision across recordings are shown, with error bars indicating the corresponding *SDs*.

Consensus analysis for the arousal detection task was performed on the 27 recording subset described in the Consensus Annotations and Human-Model Comparison section. Event-based performance of each scorer and the model was evaluated against the consensus arousal annotations using the IoU-based matching criterion and event-level metrics defined in the Model Training and Evaluation section. Table 9 summarizes recall, precision, and F1-score for individual scorers and the model, and Figure 5 shows recall and precision for each against the consensus annotations.

**Table 9:** Performance Metrics for Arousal Detection of Individual Scorers and the Model

Scorer	Recall	Precision	F_1_-score
Scorer 1	0.846 (0.062)	0.814 (0.069)	0.827 (0.047)
Scorer 2	0.746 (0.090)	0.810 (0.090)	0.770 (0.062)
Scorer 3	0.856 (0.055)	0.724 (0.091)	0.780 (0.061)
Scorer 4	0.768 (0.101)	0.819 (0.076)	0.786 (0.067)
Model	0.845 (0.083)	0.780 (0.078)	0.807 (0.056)

Values are presented as mean (*SD*) calculated from 27 recordings.

The model demonstrated comparable performance to that of human scorers.

#### Respiratory event detection consensus

**Figure 6:**
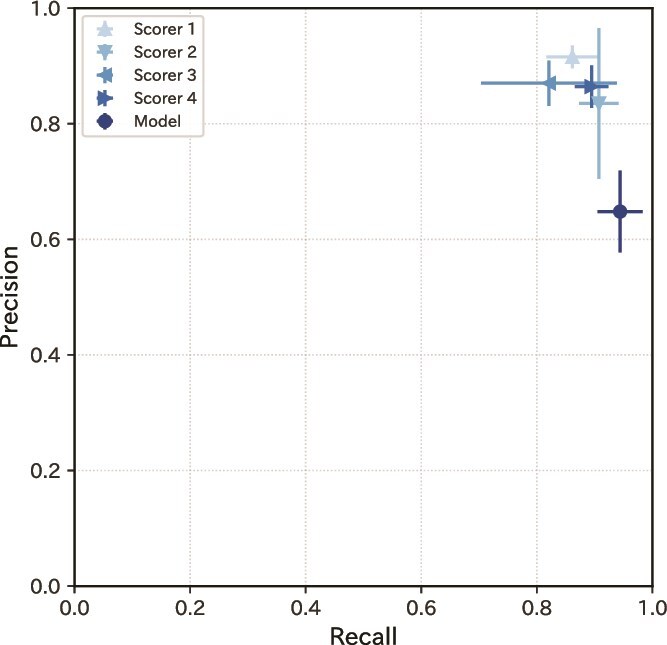
Recall and precision for respiratory event detection by individual scorers and by the model, evaluated against consensus annotations. Mean recall and precision across recordings are shown, with error bars indicating the corresponding *SDs*.

For respiratory event detection, consensus analysis was conducted on five recordings from participants with suspected SDB, as defined in the Consensus Annotations and Human-Model Comparison section. Consensus respiratory event annotations were compared with each scorer’s annotations and with the model outputs using the same IoU-based matching criterion and event-based metrics as for arousals. Table 10 summarizes recall, precision, and F1-score for individual scorers and the model, and Figure 6 illustrates recall and precision for each against the consensus annotations.

**Table 10:** Performance Metrics for Respiratory Event Detection of Individual Scorers and the Model

Scorer	Recall	Precision	F1-score
Scorer 1	0.861 (0.045)	0.916 (0.020)	0.888 (0.033)
Scorer 2	0.907 (0.034)	0.835 (0.131)	0.865 (0.078)
Scorer 3	0.821 (0.118)	0.870 (0.039)	0.842 (0.073)
Scorer 4	0.895 (0.029)	0.864 (0.037)	0.878 (0.011)
Model	0.944 (0.039)	0.648 (0.071)	0.767 (0.054)

Values are presented as mean (*SD*) calculated from five recordings.

The model demonstrated slightly lower overall performance than human scorers, characterized by higher recall and lower precision in the consensus subset.

This recall–precision imbalance contrasts with the more balanced performance observed in the Respiratory Event Detection section, where precision and recall were closely aligned. The deviation was observed specifically in the consensus subset. The consensus subset consists of five recordings, and its performance metrics therefore reflect a limited evaluation sample. In addition, these recordings were annotated at an early stage of the study, whereas most annotations were generated later under the same calibration framework. These factors may be associated with the recall–precision imbalance observed in this subset.

## Discussion

### Overview of model performances

This study evaluated three models designed for distinct sleep-related tasks: sleep stage classification, arousal detection, and respiratory event detection from PSG data. All models were implemented using GBDT architectures rather than deep learning frameworks and were designed to operate sequentially within a unified pipeline. Specifically, the arousal detection model leveraged outputs from the sleep stage classifier, and the respiratory event detection model further incorporated both sleep stage and arousal information. This hierarchical design reflects the dependency structure of physiological events during sleep and enables each downstream task to benefit from contextual information provided by preceding stages.

Detailed performance metrics for all tasks are summarized in the Performance Metrics section.

In sleep stage classification, prior work employing deep learning approaches has reported F1-scores of $0.90\ (0.04)$, $0.53\ (0.07)$, $0.85\ (0.04)$, $0.76\ (0.07)$, and $0.90\ (0.02)$ for Wake, N1, N2, N3, and REM stages, respectively [44]. In the present study, comparable performance was achieved for the Wake, N2, and REM stages, while notably higher F1-scores were observed for the N1 ($0.711\ \left[0.004\right]$) and N3 ($0.865\ \left[0.009\right]$) stages. Notably, substantial variability in N1 performance across datasets (F1-scores ranging from 0.45 to 0.64) has been documented in prior work [44], underscoring the dataset-dependent nature of this stage.

For arousal detection, previous studies have reported event-level F1-scores ranging from 0.70 to 0.79 depending on the dataset [37]. The performance achieved in the present study ($0.733\ \left[0.011\right]$) falls within this range.

With respect to respiratory event detection, earlier investigations of binary apnea–hypopnea detection have reported F1-scores between 0.517 and 0.665 [39]. In contrast, the present study achieved an F1-score of $0.818\ (0.012)$. While differences in datasets and event definitions limit direct comparability, this result indicates that robust respiratory event detection is achievable even without deep-learning-based architectures.

Taken together, these findings extend earlier observations that non-deep-learning methods can perform competitively in sleep stage classification to event-based tasks [31]. By combining interpretable GBDT classifiers with task-specific post-processing algorithms, the proposed models successfully estimated both the onset and duration of arousal and respiratory events with high temporal precision, while maintaining model interpretability and feasibility for real-world deployment. In addition, GBDT models offer advantages in terms of reproducibility, computational efficiency, and stable implementation in clinical and large-scale research environments [57, 58].

At the same time, direct quantitative comparisons across studies remain inherently challenging, as datasets, annotation protocols, and event definitions differ substantially. Accordingly, variability in reported performance across prior work highlights that achievable model accuracy is strongly influenced by dataset-specific factors, particularly the characteristics and consistency of the underlying reference annotations. For this reason, the following section focuses on a detailed examination of annotation quality and consensus scoring in the present dataset, in order to more rigorously contextualize the observed model performance.

### Annotation quality and consensus study

In this study, particular attention was paid to the quality and consistency of the reference annotations. Before scoring, all scorers participated in structured calibration sessions to align interpretation criteria and reduce self-reported variability across scorers.

As described in the Consensus Study section, the consensus analyses showed that, although performance for respiratory event detection was slightly lower than human inter-scorer agreement, the proposed models achieved human-level performance overall within the present dataset. This establishes an interpretable reference for model performance, as agreement among human experts effectively defines a practical performance ceiling under a given annotation protocol.

For sleep stage classification, a previous study using a deep-learning-based model reported F1-scores of $0.88\ (0.10)$, $0.56\ (0.14)$, $0.86\ (0.05)$, $0.73\ (0.23)$, and $0.93\ (0.05)$ for Wake, N1, N2, N3, and REM, respectively, in healthy participants, indicating performance comparable to inter-scorer agreement [44]. In the present study, the proposed model achieved comparable overall performance, while exhibiting higher performance particularly in the N1 and N3 stages. These stages are widely recognized as difficult to score consistently among human experts and are known to exhibit substantial inter-scorer variability [12–16]. The resulting differences in stage-wise performance patterns suggest that variations in annotation consistency across datasets can substantially influence achievable model performance, especially for these challenging sleep stages.

For arousal and respiratory event detection, the consensus analysis was conducted using a protocol analogous to that employed in an earlier comparative study of human and automated scoring, with a primary focus on respiratory events [45]. That study reported a model F1-score of approximately $0.57\ (0.23)$ for respiratory event detection, while human scorers achieved F1-scores in the range of 0.52–0.58. In contrast, in the present study, the model achieved an F1-score of $0.767\ (0.054)$, and human scorers achieved F1-scores ranging from 0.842 to 0.888. This higher level of agreement among human scorers was accompanied by a corresponding shift in model performance, although a gap relative to human-level performance remains. Arousal detection was not evaluated in the earlier comparative study; however, the present results similarly demonstrated comparable performance between the model and human scorers for arousal detection.

In the consensus-based comparison, respiratory event detection showed relatively high recall but lower precision, whereas the results based on the full dataset were more balanced. One possible explanation is that annotation practices may have shifted over time, such that the broader set of annotations used across model development and evaluation became relatively more permissive in identifying respiratory events than the earlier consensus subset. If so, the model would tend to detect respiratory events more readily, leading to increased false-positive detections when evaluated against the consensus reference. This pattern may reflect task-specific differences in annotation tendencies, but it does not change the broader interpretation of the results across tasks.

Across the evaluated tasks, these findings indicate that even relatively simple GBDT models can approach or achieve human-level performance. At the same time, the results indicate that higher levels of annotation quality are associated with higher achievable model performance when models are trained and evaluated on such data. This further highlights the central role of annotation quality in determining the achievable performance of automated sleep analysis systems. Moreover, explicitly characterizing human-level agreement provides an essential reference for interpreting model performance, ensuring that reported improvements are interpreted relative to the inherent variability of human scoring rather than based solely on absolute performance metrics.

### Limitations

This study has several limitations that should be considered.

One limitation is that the datasets used in this work excluded recordings with extensive signal loss, severe noise, or missing channels. While this selection ensured reliable model training and evaluation, real-world clinical PSG recordings frequently contain artifacts, partial signal loss, or reduced signal quality. In routine practice, diagnostic interpretation must therefore be performed under imperfect conditions that are not fully represented in the present datasets [59–61].

Another limitation is that differences in hardware and software among PSG systems may affect signal characteristics. Although the AASM Scoring Manual [9] provides baseline technical specifications for signal acquisition, variations in hardware configurations, sensor types, filtering pipelines, and sampling rates across PSG systems can introduce systematic differences in recorded signals [62]. In the present study, PSG data acquired at different institutions using distinct recording systems were jointly analyzed, and stable performance was observed. However, because one dataset consisted exclusively of healthy participants while the other included participants with suspected SDB, differences in participant characteristics were confounded with differences in recording systems and clinical settings. As a result, the isolated effects of hardware- or facility-related factors cannot be disentangled from population-related variability. In addition, some PSG systems have only three EEG channels. To generalize the present findings to such systems, future work may be extended to include datasets with limited EEG channels.

A further limitation concerns the annotations themselves. Although high-quality annotations were obtained through calibration sessions among four expert scorers, residual ambiguity in PSG scoring remains unavoidable. Previous studies have shown that even experienced scorers exhibit substantial inter-scorer variability and that calibration can improve agreement without fully eliminating disagreement [63]. Moreover, ambiguities in the AASM scoring rules allow room for differing interpretations. In addition, manual scoring in this study was based on Version 2.6 of the AASM Scoring Manual [50], whereas the current version is Version 3 [9]. Because Version 3 includes updates to some scoring rules, direct comparison with studies scored strictly according to the current manual should be made with caution. However, this difference is not considered to alter the main interpretation of the present study, which focuses on the effect of annotation quality on model evaluation. Inter-scorer agreement has been reported to be higher among scorers from the same clinical centers or countries than across different institutions or regions [64–66], suggesting the influence of shared local scoring conventions and institutional practices.

The calibration procedure may also have influenced the inter-scorer agreement estimates, because some of the same recordings were used both for calibration and for the consensus study, and epochs or events reviewed during calibration may have been scored more consistently thereafter. However, only a limited portion of each recording was subjected to detailed review, and most epochs or events were not directly affected by this process.

Related to the annotation quality, recent evaluations have reported that the performance of some automatic scoring systems remains insufficient for routine clinical use [67]. Such performance degradation may partly reflect mismatches in scoring conventions between training and evaluation datasets, rather than limitations of the underlying algorithms [68]. Accordingly, the present model may not achieve the same level of agreement when applied to independently annotated datasets, even when those datasets exhibit high internal inter-scorer reliability. This underscores the importance of external validation using datasets collected at different institutions and annotated by distinct scorer groups.

The study population consisted exclusively of Japanese participants. Physiological and demographic differences may influence PSG signal characteristics. In particular, skin pigmentation has been shown to affect pulse oximetry measurements [69–71]. Therefore, the generalizability of the present findings to other populations cannot be assumed, and validation in more diverse cohorts is required.

Finally, the present framework still has important scope-related limitations for comprehensive PSG interpretation. The respiratory event detection model treated all respiratory events as a single category and did not distinguish between apnea and hypopnea or between obstructive and central events, although such subtyping is clinically important for diagnosis and treatment planning [72, 73]. In addition, respiratory event detection relied primarily on the nasal pressure airflow signal and did not incorporate thermistor airflow or thoracoabdominal effort signals as model inputs. Both nasal pressure and thermistor are used in PSG to assess airflow reduction; however, in the present dataset, nasal pressure was more consistently recorded with acceptable signal quality than thermistor, and the final model was therefore developed using nasal pressure as the primary airflow input. This was considered a practical configuration for the present task, which was limited to detecting the presence or absence of respiratory events. By contrast, thoracoabdominal effort signals are particularly important for differentiating obstructive from central respiratory events, but these signals were not included because such subtype classification was outside the scope of the current model. Nevertheless, reliance on nasal pressure alone may reduce robustness when the pressure signal is degraded and may limit generalizability to datasets acquired with different airflow sensor configurations. In addition, periodic limb movements (PLM) [74], which are also an important component of comprehensive PSG interpretation alongside sleep stages, arousals, and respiratory events, were not analyzed because PLM-related annotations were not available in the current dataset. More comprehensive future development should therefore include evaluation of the complementary use of thermistor and thoracoabdominal effort signals, expansion of the analyzed channel set where appropriate, and incorporation of PLM annotation and detection.

### Clinical implications and future directions

For practical deployment, explicit handling of real-world signal artifacts is essential. Numerous studies have proposed methods for detecting artifacts in PSG signals, including approaches targeting EEG contamination, movement-related artifacts, and corruption of respiratory signals [75–78]. Such methods could be integrated into preprocessing pipelines prior to sleep staging or respiratory event detection. At the same time, there remain cases in which automated systems may struggle, whereas such cases can be readily recognized by experienced human scorers [79]. These findings further emphasize the necessity of incorporating expert review into clinical workflows.

Several studies have demonstrated that combining automated algorithms with expert review can be effective in clinical PSG workflows [80–82]. Semi-automated approaches have been shown to reduce scoring time while maintaining or improving agreement with expert consensus by allowing clinicians to focus on difficult or ambiguous segments.

Beyond signal quality, variability in PSG interpretation also arises from inter-scorer disagreement [12–16] and from local scoring conventions that develop within specific institutions or regional contexts [64–66]. Such variability complicates structured discussion of discrepant cases, as human judgments may vary across scorers and evolve over time.

In this setting, stable and reproducible model outputs may provide a useful reference for identifying scorer-related ambiguity. By comparing human judgments against consistent model predictions, ambiguous cases can be highlighted more explicitly, potentially supporting clarification and calibration of scoring decisions [83]. At the same time, models trained on institution-specific data may inherit local biases. From a practical standpoint, retraining or fine-tuning models on local datasets may allow alignment with institution-specific scoring conventions when such adaptation is clinically desirable [84, 85].

With respect to dataset size, prior work has shown that the performance of automated sleep staging models tends to plateau once the number of training recordings exceeds several hundred, with additional data contributing primarily to increased heterogeneity rather than improved accuracy [86]. The present study includes a dataset size comparable to or larger than those reported to reach this performance plateau, suggesting that the available data volume is sufficient for evaluating the proposed approach.

Importantly, further increases in dataset size may not necessarily lead to performance gains if additional data introduce greater heterogeneity in patient populations, recording systems, or annotation practices. In particular, incorporating datasets from different institutions or regions may add variability arising from local scoring conventions and implicit annotation differences, potentially reducing overall agreement. Future data expansion should therefore emphasize careful curation, harmonization of annotation practices, and strategies that explicitly address domain and annotation heterogeneity, rather than the indiscriminate aggregation of additional recordings.

In this context, approaches that explicitly characterize scoring uncertainty may further strengthen collaboration between human experts and automated systems. Methods that highlight epochs associated with intrinsically ambiguous sleep stage transitions or low model confidence can guide targeted expert review and calibration. Recent work on probabilistic and uncertainty-aware sleep staging frameworks suggests that such representations provide quantitative insight into inter-scorer disagreement and may support improved scoring consistency over time [46–48]. More broadly, recent perspectives on advanced PSG analysis have emphasized the potential of moving beyond conventional rule-based representations toward integrative frameworks that capture multidimensional sleep dynamics across time, signals, and physiological domains, thereby enabling analyses that are difficult to achieve through visual inspection alone [87].

At a system level, federated learning has emerged as a promising framework for privacy-preserving collaboration across institutions. Recent studies have demonstrated that federated approaches to sleep stage classification can achieve performance comparable to centralized training while allowing data to remain within local clinical environments [88–90]. Such strategies may facilitate large-scale validation and model refinement across institutions while mitigating data-sharing constraints.

Finally, despite these methodological advances, translating automated PSG analysis into routine clinical practice remains challenging. Prior work has highlighted practical barriers such as integration with existing clinical workflows, user trust and acceptance, interpretability of model outputs, and the need for robust validation under real-world conditions, all of which must be systematically addressed to enable safe and effective clinical adoption [91].

Taken together, the present results demonstrate that models trained on carefully curated and internally consistent PSG datasets can achieve high performance with relevance to clinical PSG analysis across multiple scoring tasks. At the same time, effective deployment in real-world clinical environments requires explicit consideration of signal artifacts, scorer-related variability, and local practice differences. Accordingly, automated PSG analysis is best implemented as part of an integrated system that supports expert review and decision-making, rather than as a standalone replacement, with continued emphasis on usability, transparency, and seamless integration into clinical workflows. In addition, practical adoption will depend on thoughtful system design that facilitates efficient interaction between automated outputs and human expertise, enabling clinicians to readily interpret, review, and refine model-assisted results in daily practice.

## Data Availability

The data underlying this article cannot be shared publicly due to ethical, legal, and contractual restrictions related to participant privacy and data ownership. The data will be shared on reasonable request to the corresponding author.
